# Exploring the genomic resources and analysing the genetic diversity and population structure of Chinese indigenous rabbit breeds by RAD-seq

**DOI:** 10.1186/s12864-021-07833-6

**Published:** 2021-07-26

**Authors:** Chenmiao Liu, Shuhui Wang, Xianggui Dong, Jiping Zhao, Xiangyang Ye, Ruiguang Gong, Zhanjun Ren

**Affiliations:** College of Animal Science and Technology, Northwest A&F University, Yangling, shaanxi China

**Keywords:** RAD-seq, Chinese indigenous rabbit breeds, Single nucleotide polymorphisms, Genomic resource, Genetic analysis

## Abstract

**Background:**

Chinese indigenous rabbits have distinct characteristics, such as roughage resistance, stress resistance and environmental adaptability, which are of great significance to the sustainable development of the rabbit industry in China. Therefore, it is necessary to study the genetic diversity and population structure of this species and develop genomic resources.

**Results:**

In this study, we used restriction site-associated DNA sequencing (RAD-seq) to obtain 1,006,496 SNP markers from six Chinese indigenous rabbit breeds and two imported rabbit breeds. Jiuyishan and Fujian Yellow rabbits showed the highest nucleotide diversity (π) and decay of linkage disequilibrium (LD), as well as higher observed heterozygosity (Ho) and expected heterozygosity (He), indicating higher genetic diversity than other rabbits. The inbreeding coefficient (*F*_IS_) of New Zealand rabbits and Belgian rabbits was higher than that of other rabbits. The neighbour-joining (NJ) tree, principal component analysis (PCA), and population structure analysis of autosomes and Y chromosomes showed that Belgian, New Zealand, Wanzai, Sichuan White, and Minxinan Black rabbits clustered separately, and Fujian Yellow, Yunnan Colourful, and Jiuyishan rabbits clustered together. Wanzai rabbits were clearly separated from other populations (K = 3), which was consistent with the population differentiation index (*F*_ST_) analysis. The selection signature analysis was performed in two populations with contrasting coat colours. With Sichuan White and New Zealand rabbits as the reference populations and Minxinan Black and Wanzai rabbits as the target populations, 408, 454, 418, and 518 genes with a selection signature, respectively, were obtained. Gene Ontology (GO) classification and Kyoto Encyclopedia of Genes and Genomes (KEGG) enrichment analysis were performed on the genes with a selection signature. The results showed that the genes with a selection signature were enriched in the melanogenesis pathway in all four sets of selection signature analyses.

**Conclusions:**

Our study provides the first insights into the genetics and genomics of Chinese indigenous rabbit breeds and serves as a valuable resource for the further effective utilization of the species.

**Supplementary Information:**

The online version contains supplementary material available at 10.1186/s12864-021-07833-6.

## Background

Rabbits (*Oryctolagus cuniculus*) are recently domesticated animals with an estimated history of approximately 1400 years [1]. In China, there are approximately 20 indigenous and recently imported rabbit breeds, mainly distributed in Sichuan, Shandong, Henan, and other provinces [2]. Over time, the Chinese indigenous rabbit has evolved features such as roughage resistance and high disease resistance, and these rabbits are widely used for meat, fur and wool [3, 4]. With the development of the market economy, a large number of foreign rabbit breeds have been imported for crossbreeding improvement and promotion in Chinese indigenous rabbits, while the protection and breeding of excellent performing indigenous rabbits have been ignored, resulting in a sharp decrease in their number [5]. Therefore, it is necessary to study the genetic diversity of Chinese indigenous rabbits and protect this breed resource.

To date, some studies have been carried out on Chinese indigenous rabbit breeds. For example, Zhao et al. set up different gradients of sample size and marker number to analyse the influence on population genetic parameters and established a genetic evaluation method for indigenous rabbit germplasm resources [6]. Additionally, Ren et al. used the genome-wide SNPs of 104 rabbits of four Chinese indigenous breeds (Sichuan White rabbit: 30, Tianfu Black rabbit: 34, Fujian Yellow rabbit: 32 and Fujian Black rabbit: 8) to systematically study their genetic diversity and population structure. The sample size of Sichuan White and Fujian Yellow rabbits was different from that of this study, and the genetic parameters and genetic distance showed obvious differences. Consistent with this study, the genetic diversity of Fujian Yellow rabbits was higher than that of Sichuan White rabbits, and Fujian Yellow and Sichuan White rabbits clustered separately [7]. However, the genetic analysis of Chinese indigenous rabbit breeds using RAD-seq has not been well studied.

The development of next-generation sequencing (NGS) technology utilizes the high-throughput detection of SNP molecular markers, providing a more efficient and accurate method for studying genetic evolution at the genome level [8]. RAD-seq technology is a simplified genome sequencing technology based on whole-genome restriction sites developed on the basis of next-generation sequencing [9]. With the development of high-throughput sequencing technology and bioinformatics technology, RAD-seq analysis has become more refined and has been applied to many organisms [10]. Zhang et al. [11] used genome-wide SNP resources generated by RAD-seq technology to develop and evaluate small yellow croaker (*Larimichthys polyactis*). Moreover, Zhai et al. [12] successfully used RAD-seq for SNP discovery and genotyping in chickens.

In this study, we discovered genome-wide SNPs in six Chinese indigenous rabbit breeds and two imported rabbit breeds using the RAD-seq method. We then investigated the genetic diversity and population structure of these breeds and developed genomic resources for them. The interbreed genetic differences revealed by this study from the perspective of autosomes and Y chromosomes help us to better establish conservation strategies for genetic diversity and crossbreeding systems in the rabbit industry. Furthermore, the SNP dataset generated in this study provides a valuable resource for future genetics and genomics research in this species.

## Results

### RAD-tag sequencing and data filtering

We obtained 160.20G of raw data by Illumina sequencing, with an average of 2.19G per sample, ranging from 0.73 to 5.00G. After quality filtering of the sequence data, 158.79G of clean data (0.72G to 4.94G for each sample, with an average of 2.18G) were retained. Of the clean reads retained, an average of 14.79 million reads were retained for each sample. The Q20 was higher than 96.13%, the Q30 was higher than 90.64%, and the GC content was stable between 40.91 and 59.00% (Supplementary Table S1). Overall, our sequencing data showed a high Phred quality.

### Population genetic diversity

**Fig. 1:**
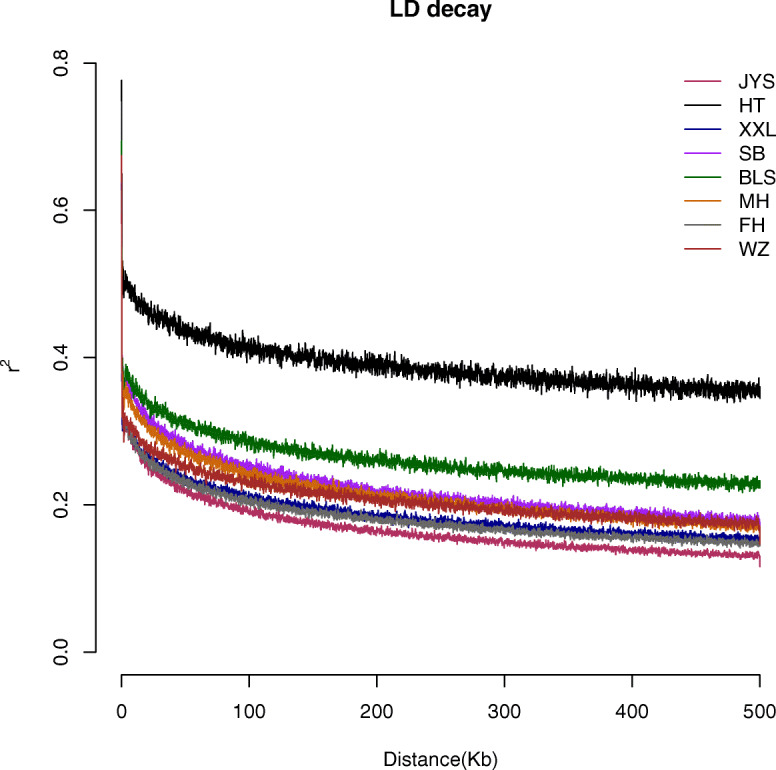
LD attenuation map of 6 Chinese indigenous rabbit breeds and 2 imported rabbit breeds

There were differences in the number of SNPs between the six Chinese indigenous rabbit breeds and the two imported rabbit breeds, and the number of SNPs was recorded in the following order: Jiuyishan rabbits > Fujian Yellow rabbits > New Zealand rabbits > Sichuan White rabbits > Minxinan Black rabbits > Wanzai rabbits > Belgian rabbits > Yunnan Colourful rabbits. Jiuyishan and Fujian Yellow rabbits had the largest number of SNPs. The nucleotide diversity of each breed was estimated from the SNP data. Because nucleotide diversity represents genetic diversity to an extent, it can be concluded from Table 1 that the nucleotide diversity of Jiuyishan and Fujian Yellow rabbits was the highest. The LD attenuation analysis showed that the LD coefficient attenuation rate of different breeds was different. Jiuyishan and Fujian Yellow rabbits exhibited a rapid decay rate and a low level of LD (Fig. 1). The Ho and He of Jiuyishan and Fujian Yellow rabbits were higher than those for other rabbits. The *F*_IS_ of New Zealand and Belgian rabbits was at a high level, and the *F*_IS_ of New Zealand rabbits reached 0.3256 (Table 1).

**Table 1:** Comparison of population genetic parameters in six Chinese indigenous rabbit breeds and two imported rabbit breeds

Breed	N	π	Ho	He	Average_Fis	Average_***F***_**ST**_
Sichuan White Rabbit	629,467	0.00009980	0.2137	0.2805	0.2382	0.1433
New Zealand Rabbit	631,023	0.0001012	0.1917	0.2843	0.3256	0.1431
Belgian Rabbit	581,987	0.00009590	0.2005	0.2619	0.2345	0.1562
Jiuyishan rabbit	695,290	0.0001128	0.2470	0.3178	0.2226	0.09439
Wanzai Rabbit	606,627	0.00009320	0.1988	0.2617	0.2403	0.1696
Yunnan colorful Rabbit	533,939	0.00009740	0.2261	0.2464	0.08230	0.1524
Min xi’nan Black Rabbit	621,517	0.00009620	0.2151	0.2698	0.2026	0.1524
Fujian Yellow Rabbit	670,654	0.0001096	0.2255	0.3072	0.2660	0.1083

*N* Number of SNPs, *π* Nucleotide diversity, *Ho* Observed heterozygosity, *He* Expected heterozygosity; *F*_IS_, inbreeding coefficient; *F*_ST_, population differentiation index

### Population genetic structure

**Fig. 2:**
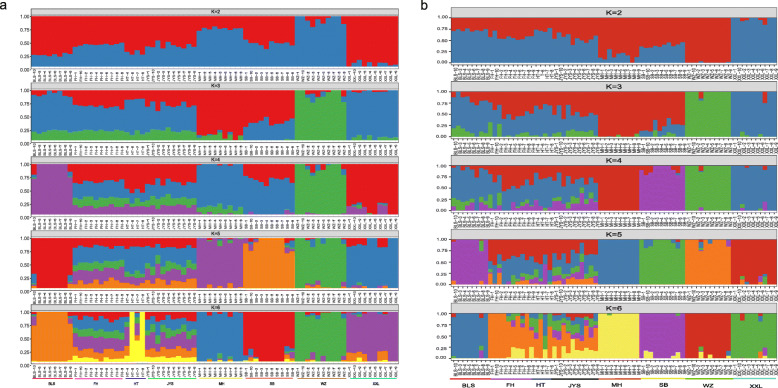
Groups structure clustering figure of 6 Chinese indigenous rabbit breeds and 2 imported rabbit breeds. **a** Structure analyses based on autosome. **b** Structure analyses based on Y- chromosome

**Fig. 3:**
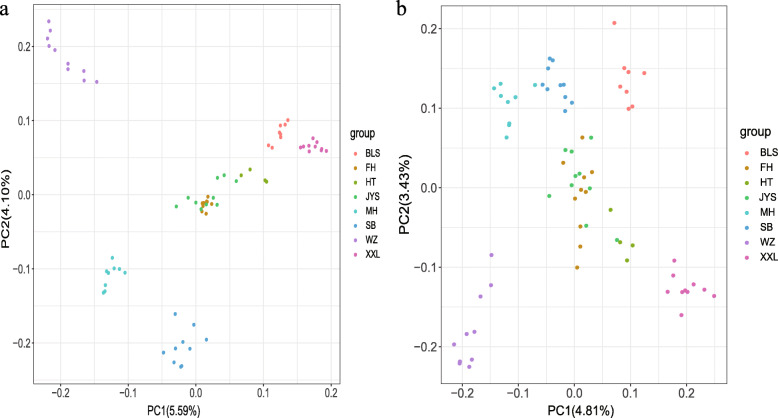
Principal components analysis of 6 Chinese indigenous rabbit breeds and 2 imported rabbit breeds. **a** PCA analyses based on autosome (PC1 = 5.59%, PC2 = 4.10%). **b** PCA analyses based on Y- chromosome (PC1 = 4.81%, PC2 = 3.43%)

**Fig. 4:**
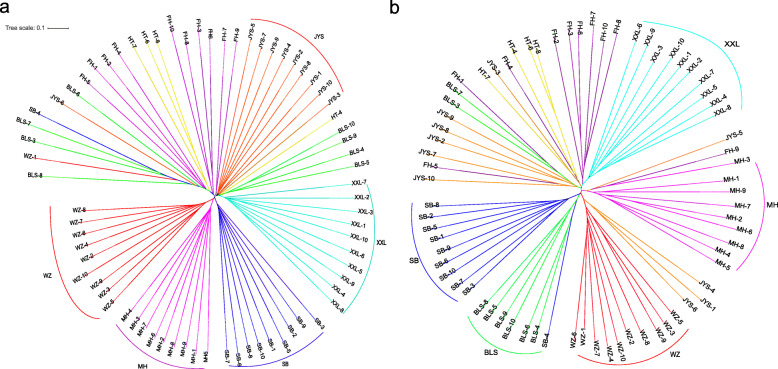
Phylogenetic tree construction with the neighbor-joining (NJ) method. **a** NJ tree based on autosome. **b** NJ tree based on Y- chromosome

Genetic analysis of population structure using ADMIXTURE software and PCA showed similar patterns. At K = 5 and K = 6, Belgian, New Zealand, Wanzai, Sichuan White, and Minxinan Black rabbits clustered separately, and their ancestral background was relatively pure, with a single major genetic ancestor. Fujian Yellow, Yunnan Colourful, and Jiuyishan rabbits grouped together and had multiple genetic ancestors. At K = 3, Wanzai rabbits were obviously separated from other rabbit breeds, indicating that this breed was phylogenetically distant from other rabbit breeds (Fig. 2). Based on the first two PCs (PC1 = 5.59%, PC2 = 4.10%), we found that Belgian, New Zealand, Wanzai, Sichuan White, and Minxinan Black rabbits clustered separately, and Fujian Yellow, Yunnan Colourful, and Jiuyishan rabbits grouped together (Fig. 3). This is consistent with the results obtained at other PC levels (Supplementary Figs. S1 and S2). The phylogenetic tree constructed by the NJ method showed that Wanzai, Minxinan Black, and Sichuan White rabbits had a close genetic relationship, forming an independent branch (Fig. 4). The *F*_ST_ values were calculated to study the genetic distance between different breeds. As shown in Table 1, it can be concluded that the average *F*_ST_ between Wanzai rabbits and other rabbit breeds was 0.1696, which was the highest among all averages, indicating a large genetic distance between Wanzai rabbits and other rabbit breeds; these findings were consistent with the results of population structure analysis.

The phylogenetic tree, PCA and population structure analysis (K = 5) constructed by Y-chromosome SNPs showed that Belgian, New Zealand, Wanzai, Sichuan White, and Minxinan Black rabbits clustered separately, and Fujian Yellow, Yunnan Colourful, and Jiuyishan rabbits grouped together. At K = 3, Wanzai rabbits were obviously separated from other rabbit breeds (Figs. 2, 3 and 4). Overall, the population structure analysis using autosomes and Y chromosomes data showed similar results.

### Inference of effective population size history

**Fig. 5:**
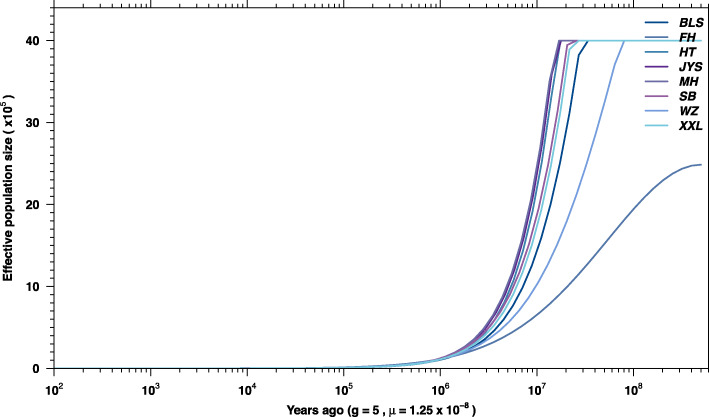
Effective population size analysis results for 6 Chinese indigenous rabbit breeds and 2 imported rabbit breeds

The eight rabbit breeds exhibited concordant demographic trajectories, with an apparent decline. Since tens of millions of years ago, the effective population size has gradually decreased. The effective population size of Fujian Yellow rabbits was always lower than that of the other seven breeds. The effective population size of most rabbits decreased sharply 2 × 10^7^–8 × 10^7^ years ago. The effective population size of Wanzai rabbits decreased sharply earlier, 6.4 × 10^7^–8.0 × 10^7^ years ago (Fig. 5).

### Analyses of the selection signatures

**Fig. 6:**
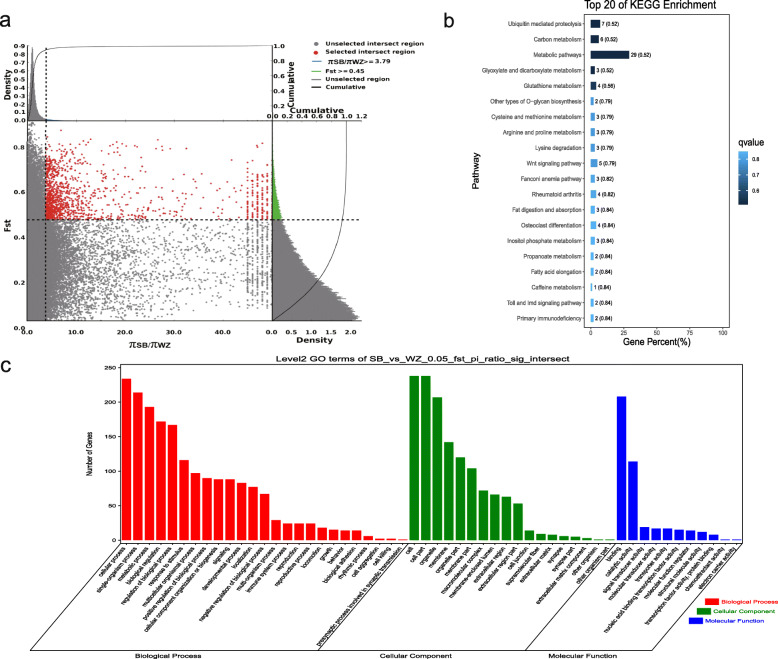
GO classification and KEGG enrichment of the selected genes of Wanzai rabbits (SB_vs_WZ). **a** Sichuan White rabbits were the control group and Wanzai rabbits were the selection group; 454 selected genes were obtained. **b** KEGG enrichment of the selected genes of Wanzai rabbits. **c** GO classification of the selected genes of Wanzai rabbits

**Fig. 7:**
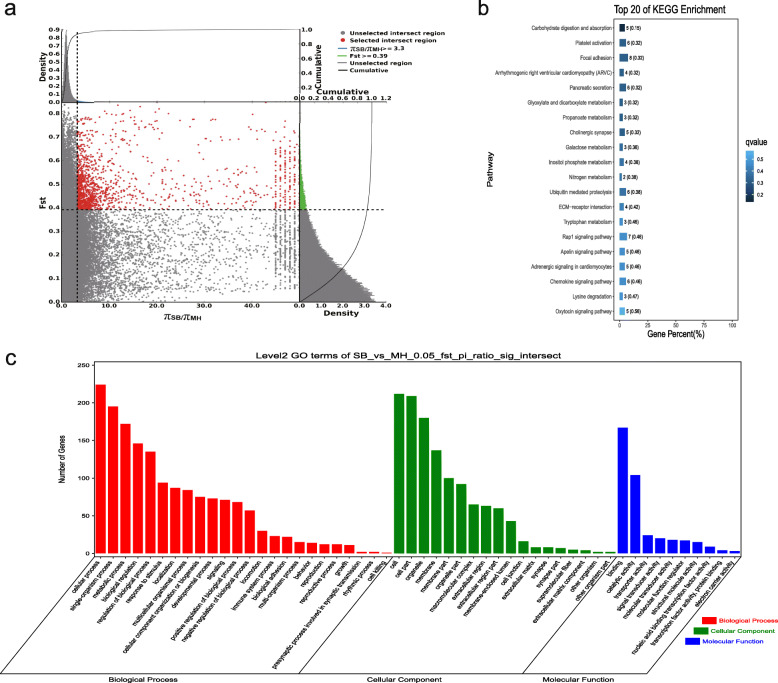
GO classification and KEGG enrichment of the selected genes of Minxinan Black rabbits (SB_vs_MH). **a** Sichuan White rabbits were the control group and Minxinan Black rabbits were the selection group; 408 selected genes were obtained. **b** KEGG enrichment of the selected genes of Minxinan Black rabbits. **c** GO classification of the selected genes of Minxinan Black rabbits

**Fig. 8:**
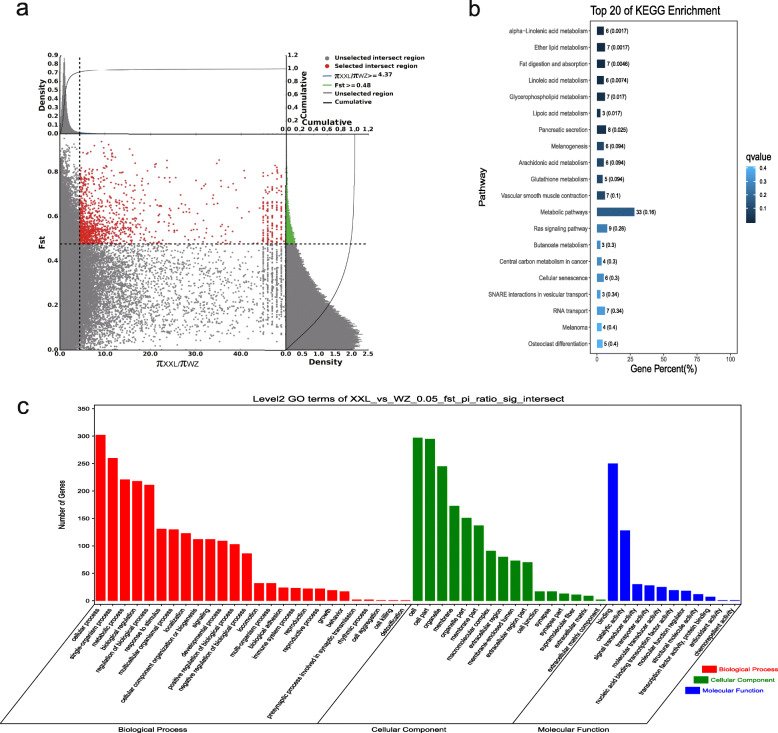
GO classification and KEGG enrichment of the selected genes of Wanzai rabbits (XXL_vs_WZ). **a** New Zealand rabbits were the control group and Wanzai rabbits were the selection group; 518 selected genes were obtained. **b** KEGG enrichment of the selected genes of Wanzai rabbits. **c** GO classification of the selected genes of Wanzai rabbits

**Fig. 9:**
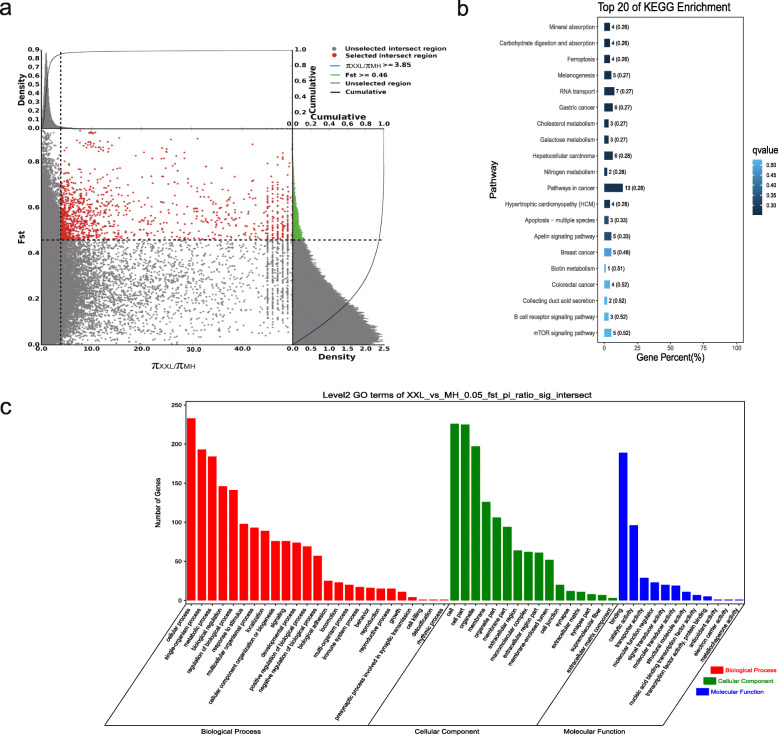
GO classification and KEGG enrichment of the selected genes of Minxinan Black rabbits (XXL_vs_MH). **a** New Zealand rabbits were the control group and Minxinan Black rabbits were the selection group; 418 selected genes were obtained. **b** KEGG enrichment of the selected genes of Minxinan Black rabbits. **c** GO classification of the selected genes of Minxinan Black rabbits

*F*_ST_ and the π ratio were used to select the top 5% of regions. With Sichuan White rabbits as the control group and Wanzai rabbits as the selection group, 454 genes with a selection signature were obtained. Among them, two genes with a selection signature (*Asip* and *Plcb4*) were related to melanogenesis. With Sichuan White rabbits as the control group and Minxinan Black rabbits as the selection group, 408 genes with a selection signature were obtained. Among them, two genes with a selection signature (*Asip* and *Plcb4*) were related to melanogenesis. With New Zealand rabbits as the control group and Wanzai rabbits as the selection group, 518 genes with a selection signature were obtained. Among them, six genes with a selection signature (*Map 2 k1*, *Edn1*, *Mitf*, *Gnai1*, *Asip*, and *Wnt3*) were related to melanogenesis. With New Zealand rabbits as the control group and Minxinan Black rabbits as the selection group, 418 genes with a selection signature were obtained. Among them, five genes with a selection signature (*Wnt10a*, *Kitlg*, *Wnt6*, *Mitf*, and *Asip*) were related to melanogenesis (Figs. 6, 7, 8 and 9, Supplementary Table S2, S3, S4, S5).

**Fig. 10:**
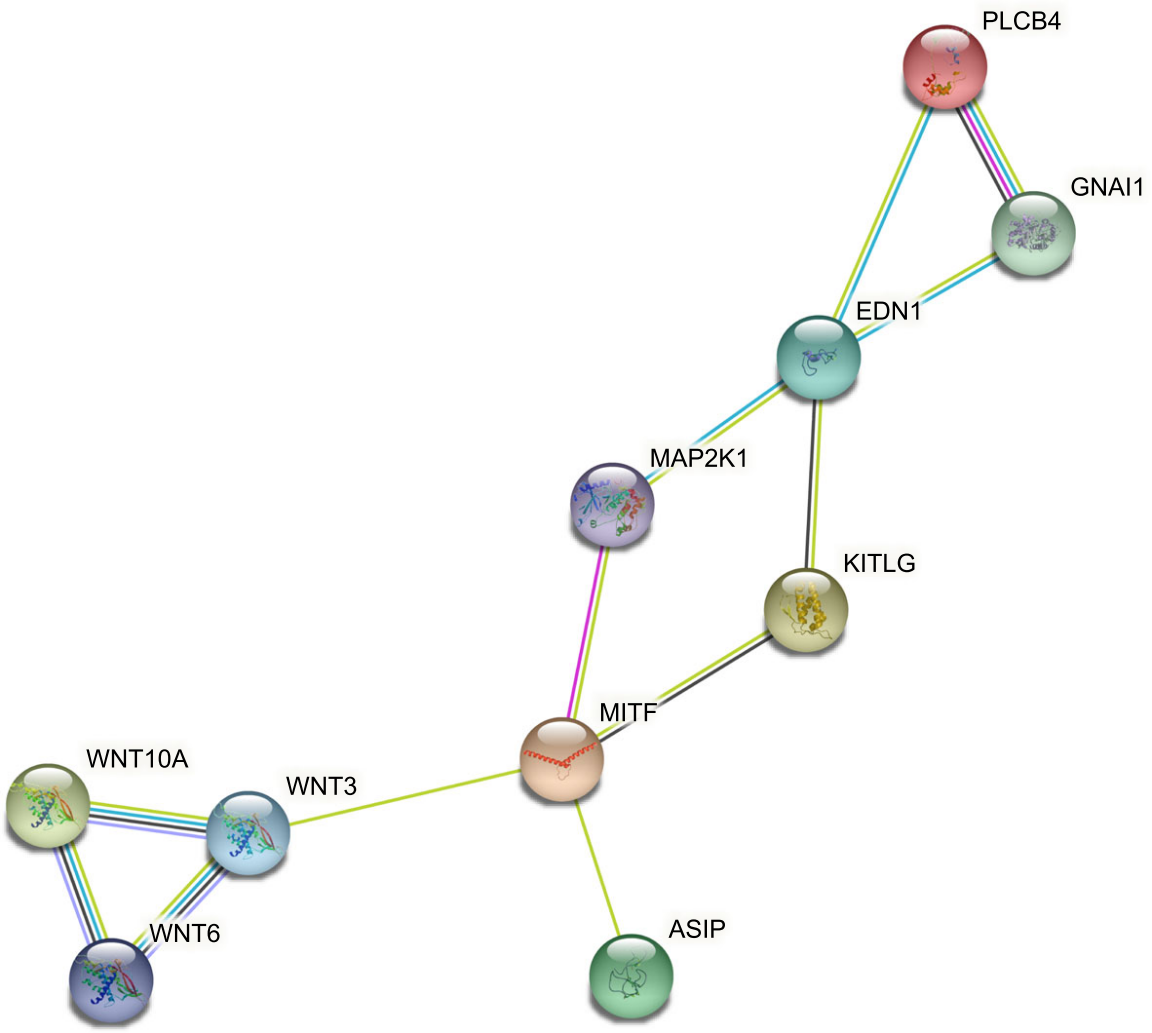
The PPI network of gene expression proteins involved in melanogenesis

A total of ten genes related to melanogenesis were obtained. A PPI network of the products of the ten genes, constructed with STRING (https://string-db.org/), showed that there was an interaction between five encoded proteins (*Plcb4*, *Gnai1*, *Edn1*, *Map 2 k1*, and *Kitlg*). There was an additional interaction between another five encoded proteins (*Map 2 k1*, *Kitlg*, *Mitf*, *Wnt3*, and *Asip*). Finally, *Wnt6*, *Wnt3*, and *Wnt10a* interacted and were located in the Wnt signalling pathway (Fig. 10).

### GO and pathway enrichment analyses

GO classification was carried out on the genes with a selection signature of Wanzai rabbits and Minxinan Black rabbits. In the four sets of selection signature analyses, cellular process (GO:0009987), single-organism process (GO:0044699), and metabolic process (GO:0008152) were the most abundant biological process subcategories. Cell (GO:0005623), cell part (GO:0044464), and organelle (GO:0043226) were the most abundant cellular component categories. Binding (GO:0005488) and catalytic activity (GO:0003824) represented the main molecular functions (Figs. 6, 7, 8 and 9, Supplementary Tables S6, S7, S8 and S9).

KEGG enrichment analysis of the genes with a selection signature of Wanzai rabbits and Minxinan Black rabbits was performed. In the four sets of selection signature analyses, the genes with a selection signature were enriched in the melanogenesis pathway, and genes related to melanogenesis were identified (Figs. 6, 7, 8 and 9, Supplementary Tables S10, S11, S12 and S13).

## Discussion

In this study, we used RAD-seq to detect 1,006,496 SNP markers from six Chinese indigenous rabbit breeds and two imported rabbit breeds. The filtered SNPs were subjected to LD attenuation analysis and nucleotide diversity analysis (π). The results indicated that Jiuyishan and Fujian Yellow rabbits had the highest genetic diversity. This may be due to crossbreeding with introduced foreign breeds [13, 14]. According to the Ho and He values, Jiuyishan and Fujian Yellow rabbits had more genetic variation and abundant genetic diversity. Among them, the Ho of Yunnan Colourful rabbits was higher, which was consistent with its diversified phenotype [15]. The *F*_IS_ of New Zealand and Belgian rabbits was higher than that of Chinese indigenous rabbit breeds, which may be due to the small initial population size of imported breeds. In the conservation process of imported breeds, the rapid increase in inbreeding should be strictly controlled to maintain the relative stability of genetic diversity. Population structure analysis and PCA showed that Belgian, New Zealand, Wanzai, Sichuan White, and Minxinan Black rabbits had better conservation efficiency than the other breeds, and Fujian Yellow, Yunnan Colourful, and Jiuyishan rabbits were mixed breeds with a very similar genetic composition: these features may be attributable to crossbreeding between Fujian Yellow, Yunnan Colourful, and Jiuyishan rabbits and the same introduced foreign breeds [13–15]. Population structure analysis and *F*_ST_ results suggested that Wanzai rabbits were phylogenetically distant from other rabbit breeds; this may be due to the geographical distribution of the Wanzai rabbits. Wanzai County is dominated by low mountains and hills, and its geographical location is remote. Wanzai rabbits have been subjected to self-rearing and closed breeding in this environment for a long time [16]. The effective population size of the eight rabbit breeds showed an apparent decline, which was most likely the result of the mass biotic extinction events caused by an asteroid hitting Earth 6.5 × 10^7^ years ago. This decline may also be due to the sharp decrease in global temperature and the extinction of terrestrial organisms to varying degrees during the transition from the Eocene to the Oligocene (3.4 × 10^7^ years ago) [17]. The genes with a selection signature of Wanzai and Minxinan Black rabbits were identified by selection signature analysis. GO classification and KEGG enrichment analysis were performed on the identified genes with a selection signature, and genes related to melanogenesis were found. Overall, our findings established interesting targets for genetic association analysis of the coat colour traits of Chinese indigenous rabbit breeds and provided a basis for additional genome studies in this species [18, 19].

The genes associated with melanogenesis in Chinese indigenous rabbit breeds were further analysed. There were interactions between *Asip*, *Map 2 k1*, *Kitlg*, *Wnt3*, and *Mitf*. *Asip* is associated with the formation of tyrosine and melanin, thus regulating coat colour [20]. *Mitf* is an important factor in the development and maturation of pigment cells and participates in the regulation of melanogenesis, thereby affecting the coat colour of rabbits [21]. *Plcb4*, *Gnai1*, *Map 2 k1*, *Kitlg,* and *Edn1* interact and affect the coat colour of rabbits by the PKA signalling pathway. The phosphorylation reaction mediated by the cAMP-PKA signalling pathway plays an important role in the process of melanogenesis. Activated PKA ultimately activates the expression of *Tyr*, thereby promoting melanogenesis [22]. The Wnt signalling pathway (*Wnt6*, *Wnt3*, and *Wnt10a*) plays an important role in the induction and differentiation of neural crest stem cells and the development of melanocytes, thereby affecting the production of melanin [23, 24].

In summary, we reported the exploration of tens of thousands of SNPs to examine the genetic relationship from the perspective of autosomes and Y chromosomes, and we compared the genetic diversity of Chinese indigenous rabbit breeds and imported rabbit breeds. The genomic resources related to coat colour of Chinese indigenous rabbit breeds were explored by selection signature analysis. These findings will help us better protect and develop the genetic resources of the species and provide a valuable resource for subsequent gene linkage and association analyses.

## Conclusions

We successfully employed RAD-seq to explore the genetic diversity and population structure of Chinese indigenous rabbit breeds and imported rabbit breeds, and we identified the genes related to melanogenesis. We found that Jiuyishan and Fujian Yellow rabbits had the highest genetic diversity; Belgian, New Zealand, Wanzai, Sichuan White, and Minxinan Black rabbits had better conservation efficiency, while Fujian Yellow, Yunnan Colourful, and Jiuyishan rabbits had multiple genetic ancestors. Because of its remote geographical location, Wanzai rabbits were phylogenetically distant from other rabbit breeds. These findings are beneficial to the resource conservation of Chinese indigenous rabbit breeds and provide a certain direction for future breeding work.

## Methods

### Sample preparation and genomic DNA extraction

The rabbit breeds were sampled from six places, namely, Sichuan, Hunan, Jiangxi, Yunnan and Fujian provinces in China. All rabbits used in the study were obtained from local conservation farms. A total of 71 blood samples from male rabbits from eight populations were collected, and each sample was derived from a different family; there was no kinship among the individuals (Table 2). In this study, we used the ear vein to collect blood without anaesthetizing the rabbits. The volume of each sample was 5 ml, and the animals sampled were released. The steps were as follows: (1) placing the rabbits in the fixed box and removing the hair from the blood collection site, (2) wiping the ear shell with xylene cotton balls to dilate the blood vessels in the ear, (3) using a blade to make a small cut in the blood vessel to allow the blood to flow out naturally, and (4) collecting the blood and pressing the wound with cotton balls to stop the bleeding. DNA samples were extracted following the CTAB method. Then, Qubit (Thermo Fisher Scientific, Waltham, MA, USA) and Nanodrop (Thermo Fisher Scientific, Waltham, MA, USA) were used to evaluate the quality of the DNA [25].

**Table 2:** Information for six Chinese indigenous rabbit breeds and two imported rabbit breeds

Number	Breed	Code	Sampling site	Fur color	Sample size	Longitude	Latitude
**1**	Sichuan White Rabbit	SB	Chengdu, Sichuan	White	10	104.06	30.67
**2**	New Zealand Rabbit	XXL	Chengdu, Sichuan	White	10	104.06	30.67
**3**	Belgian Rabbit	BLS	Chengdu, Sichuan	Tawny, Grizzle	8	104.06	30.67
**4**	Jiuyishan rabbit	JYS	Ningyuan, Hunan	White, Gray	10	111.93	25.60
**5**	Wanzai Rabbit	WZ	Wanzai, Jiangxi	Black	10	114.43	28.12
**6**	Yunnan colorful Rabbit	HT	Dali, Yunnan	Hemp gray, White, Black	4	100.23	25.60
**7**	Min xi’nan Black Rabbit	MH	Shanghang, Fujian	Black	9	116.42	25.05
**8**	Fujian Yellow Rabbit	FH	Lianjiang, Fujian	Yellow, White	10	119.53	26.20
**Total**					71		

### Library construction and Illumina sequencing

According to a modified protocol for building RAD-seq libraries [26]. In general, EcoRI (New England Biolabs) was used to digest the whole genome, and P1 adapters were ligated to their cut sites. The samples were then collected, randomly cut, and size selected. After adding the P2 adapters, DNA fragments ranging from 300 to 700 bp were used to construct the sequencing libraries. Finally, the samples were sequenced on the Illumina HiSeq 3000 platform (Illumina, San Diego, California, USA) using 100-bp paired-end reads.

### Data processing and SNP calling

The generated reads were filtered using the fastp tool (v0.19.5) [27]. According to the following three stringent filtering standards, raw reads were processed into high-quality clean reads: (1) remove reads with greater than or equal to 10% unidentified nucleotides (N), (2) remove reads with greater than 50% bases and Phred quality scores less than or equal to 20, and (3) remove reads aligned with barcode adapters. The filtered clean reads were used for assembly analysis.

Burrows-Wheeler Aligner software was used to align the clean reads of each sample with the reference genome (https://www.ncbi.nlm.nih.gov/genome/?term=rabbit), with the following parameters “mem 4 -k 32 -M”, where -k is the minimum seed length, and -M is an option to mark shorter split alignment hits as secondary alignments [28]. Variant calling was conducted using GATK’s Unified Genotyper [29]. SNPs were filtered using GATK’s Variant Filtration with appropriate standards (−Window 4, −filter “QD < 2.0 || FS > 60.0 || MQ < 40.0 “, −G_filter “GQ < 20”) [7]. Finally, the obtained SNPs were filtered with VCFtools (https://github.com/vcftools/vcftools) for further analysis with a minor allele frequency (MAF) > 0.05 and a proportion of missing genotyping data < 20% as parameters. This data file was then used in subsequent analyses [30].

### Reassembly of Y chromosomes RAD tags

After clean reads were aligned to autosomes, X chromosomes, mitochondria, and scaffolds, the unaligned reads of the reference genome were classified as Y chromosomes sequences and reassembled. First, stack (version 1.46) was used to cluster read1 of all individuals separately to obtain individual stacks [31]. Subsequently, the stacks among individuals were clustered to obtain the stack set of the population. On the basis of the clustering results of read1, read2 was classified, and then read2 was spliced. The Y chromosomes were constructed by connecting the stacks obtained from read1 splicing and the contigs obtained from read2 splicing. These data were used as a reference sequence for subsequent mutation detection and advanced analysis. SNP calling was performed after Y chromosomes assembly.

### Population genetics analyses

First, VCFtools software was used to study the overall read depth and chromosome distribution of all SNPs [30]. The minimum read coverage for a SNP to be called was 3×, and all polymorphic loci with non-completely missing (−max-missing 1e-06-non-ref-af 1e-06) were used for counting. The nucleotide diversity (π), expected heterozygosity (He) and observed heterozygosity (Ho) of each breed were calculated by the PopGenome software package (https://cran.r-project.org/web/packages/PopGenome/vignettes/Whole_genome_analyses_using_VCF_files.pdf?tdsourcetag=s_pctim_aiomsg) [32]. In addition, PopLDdecay was used to estimate the LD attenuation trend by calculating the LD coefficient (r^2^) between two points in a range of sequences (typically< 5 Mb) [33]. The faster r^2^ decays, the higher the population genetic diversity [34]. The *F*_IS_ value was calculated using PopGen32 software [35]. After obtaining the *F*_IS_ value for each sample, the average value within the population was determined.

### Population structure analyses

After identifying SNPs, 1,006,496 SNPs were used for phylogenetic tree construction, principal component analysis and population structure analysis to clarify the evolutionary relationship between rabbit breeds. The phylogenetic tree was constructed using TreeBeSTv1.9.2 software (http://treesoft.sourceforge.net/treebest.shtml) to determine the evolutionary relationship between breeds [36]. GCTA software was used for PCA to obtain the principal component value of each sample, and then R packages were used to draw PCA scatter plots to further study the population genetic structure [37]. In structure analysis, the program ADMIXTURE Version 1.3.0 (http://software.genetics.ucla.edu/admixture/download.html) was used to infer the population structure. For each of the different subgroups (K = 2–6), the population classification and the ancestry composition of each individual were simulated. *F*_ST_ was calculated with PopGenome software to study the genetic distance between different breeds [32]. After the SNPs from the Y chromosomes alignment were filtered with a missing rate of > 0.4, the remaining 4777 SNPs were used for phylogenetic tree construction, PCA and population structure analysis with the same methods described above.

### Effective population size history analyses

We used the pairwise sequentially Markovian coalescent (PSMC) to infer historical changes in effective population size based on a single fully resequenced diploid individual [38]. In addition, we implemented the SMC++ method, which can infer the effective population size history from hundreds of individuals and is more powerful than PSMC at recovering the history for very short time scales [39]. For PSMC/SMC++ analysis, scaling was performed using a neutral mutation rate μ = 1.25 × 10^− 8^ and a generation time of 5 years. We selected 71 samples with the highest sequencing depth for SMC++ analysis, and the parameters were set to -p 0.5 -m 2.5e-8 - w 100 -em 20 -sp cubic.

### Analyses of the selection signatures

In this study, we mainly explored the genomic resources related to coat colour. Breeds were divided into two groups based on coat colour. With Sichuan White and New Zealand rabbits as the reference populations and Minxinan Black and Wanzai rabbits as the target populations, the genes of Wanzai and Minxinan Black rabbits that were under selection were identified. 100-kb windows and 10-kb steps were used for selection signature detection. The lower end of the diversity windows was selected using the -log10 transform of Nei’s π. These parameters were quantified by internal PERL scripts. The top 5% *F*_ST_ values and π ratios were selected as candidate regions [40]. The related graphs were drawn using R scripts [41]. Candidate genes in the sweep regions were extracted for further analysis.

### GO enrichment analyses and pathway enrichment analyses

GO enrichment analysis was performed using WEGO software (http://wego.genomics.org.cn/). The number of genes associated with each term was calculated [42]. Pathway-based analysis is helpful to further understand the biological function of genes. KOBAS software (http://kobas.cbi.pku.edu.cn/anno_iden.php) and the KEGG database (http://www.genome.jp/kegg/) were used to test the statistical enrichment of the genes with a selection signature [43]. The calculated *p-*values were corrected for the false discovery rate (FDR), and an FDR threshold less than or equal to 0.05 was applied. The pathway meeting this condition was defined as the pathway of significant enrichment.

## Supplementary Information

**Additional file 1: Supplementary Figure S1.** Principal component analysis (PCA) of the Chinese indigenous rabbit breeds based on autosome (PC1=5.59%, PC3=3.41%).

**Additional file 2: Supplementary Figure S2.** Principal component analysis (PCA) of the Chinese indigenous rabbit breeds based on autosome (PC2=4.10%, PC3=3.41%).

**Additional file 3: Supplementary Table S1.** Sequencing results and quality filtering of reads.

**Additional file 4: Supplementary Table S2-S5.** The results of the selection signature analysis.

**Additional file 5: Supplementary Table S6-S9.** GO classification of the selected genes in Wanzai rabbits and Minxinan Black rabbits.

**Additional file 6: Supplementary Table S10-S13.** KEGG enrichment of the selected genes in Wanzai rabbits and Minxinan Black rabbits.

## Data Availability

The sequence data have been deposited in NCBI SRA repository under accession number PRJNA544811. Reference genome sequences were downloaded from the NCBI genome assembly website for rabbit (https://www.ncbi.nlm.nih.gov/genome/?term=rabbit).

## Abbreviations

RAD-seq: Restriction site-associated DNA sequencing
π: Nucleotide diversity
LD: Linkage disequilibrium
Ho: Observed heterozygosity
He: Expected heterozygosity
*F*_IS_: Inbreeding coefficient
NJ: Neighbor-joining
PCA: Principal component analysis
*F*_ST_: Population differentiation index
GO: Gene Ontology
KEGG: Kyoto Encyclopedia of Genes and Genomes
SSR: Simple sequence repeat
NGS: Next-generation sequencing
BWA: Burrows-Wheeler Aligner
FDR: False discovery rate
