# Supplementary figures and images for: Exploring the genomic resources and analysing the genetic diversity and population structure of Chinese indigenous rabbit breeds by RAD-seq
Source: BMC Genomics. 2021 Jul 26;22:573. doi: 10.1186/s12864-021-07833-6 (PMC8314496; doi:10.1186/s12864-021-07833-6)

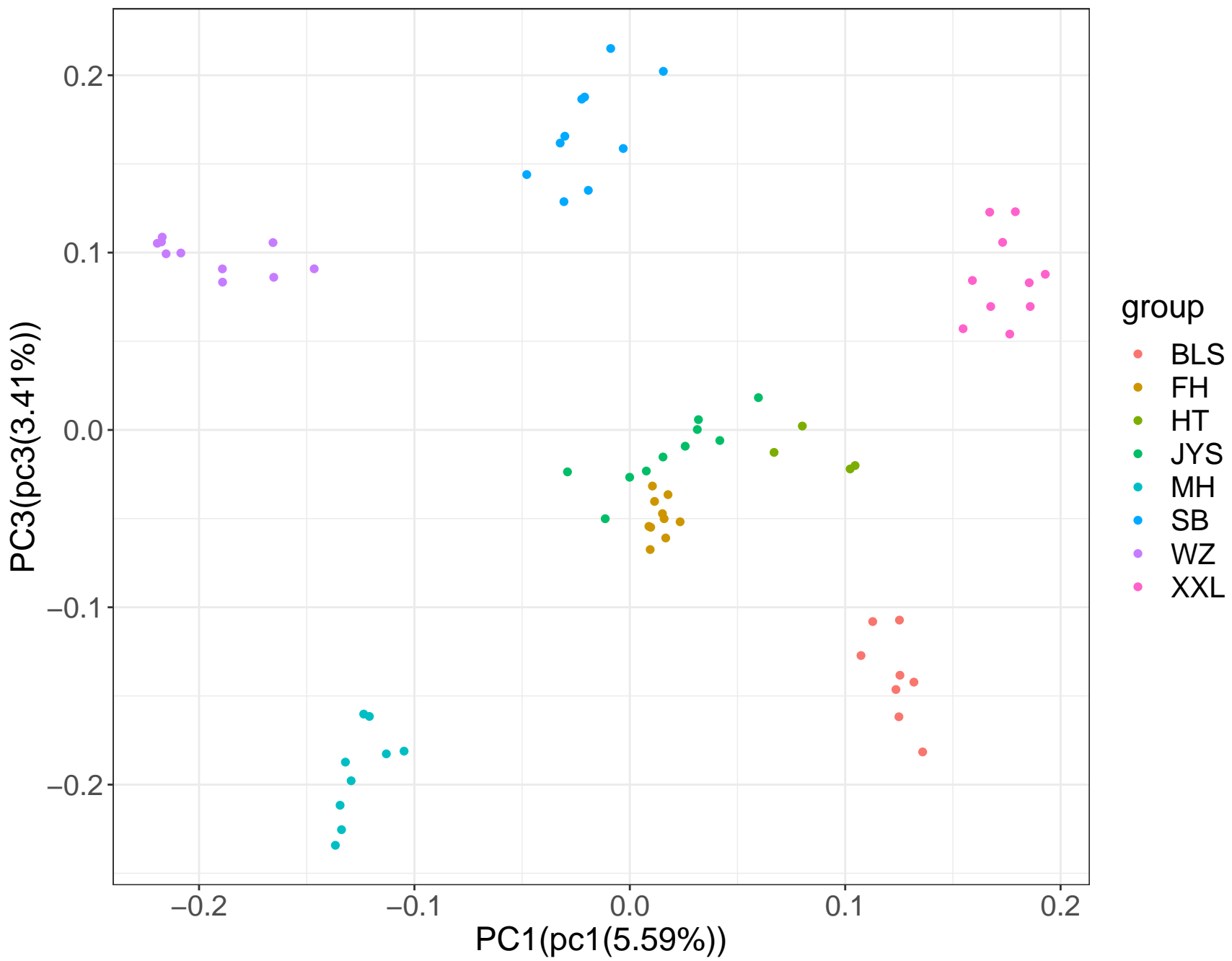

Supplement: Supplementary file 1 — Additional file 1: Supplementary Figure S1. Principal component analysis (PCA) of the Chinese indigenous rabbit breeds based on autosome (PC1=5.59%, PC3=3.41%). [file 12864_2021_7833_MOESM1_ESM.pdf]

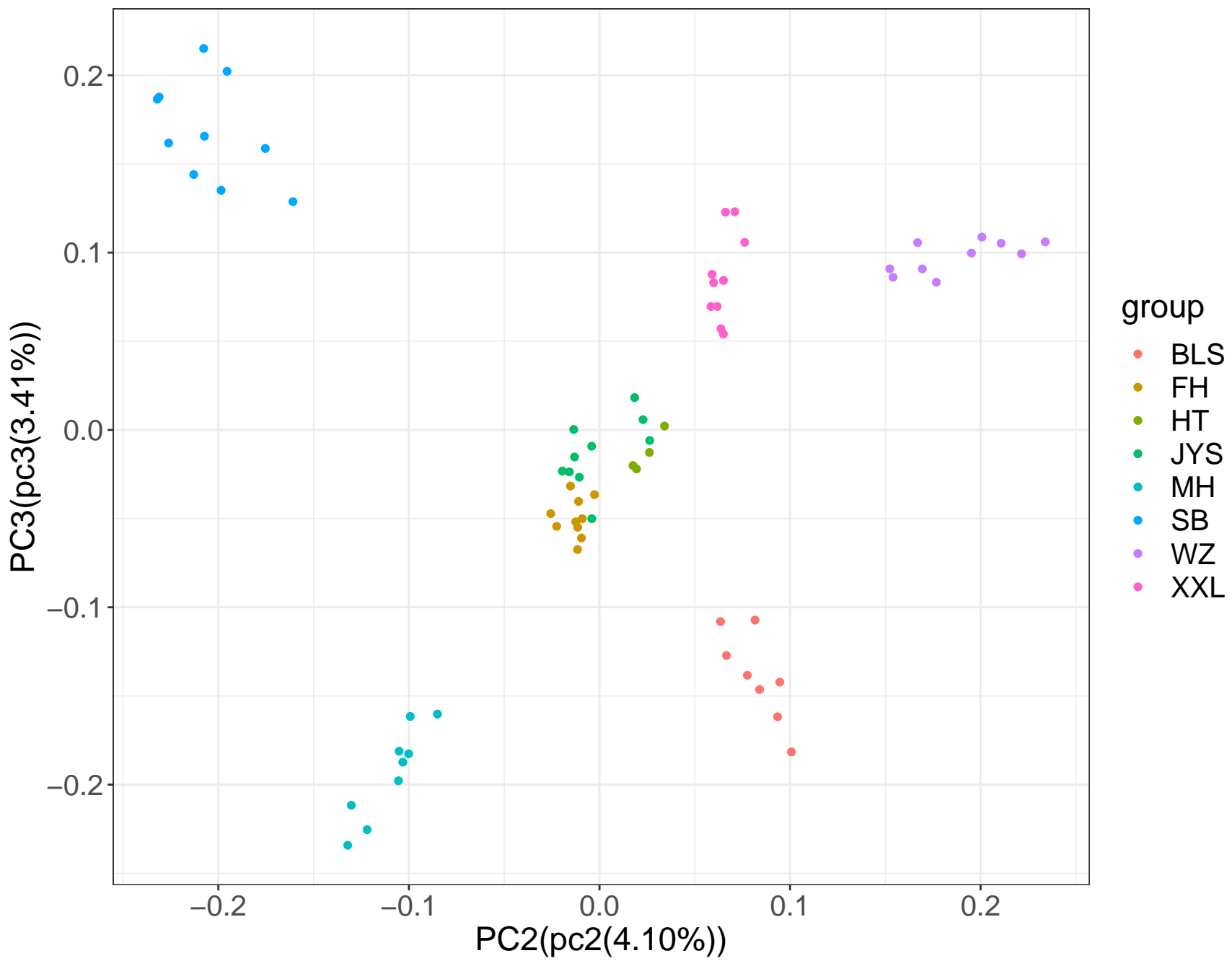

Supplement: Supplementary file 2 — Additional file 2: Supplementary Figure S2. Principal component analysis (PCA) of the Chinese indigenous rabbit breeds based on autosome (PC2=4.10%, PC3=3.41%). [file 12864_2021_7833_MOESM2_ESM.pdf]
